# Multifunctional Polydopamine-Based Nanoparticles for Dual-Mode Imaging Guided Targeted Therapy of Lupus Nephritis

**DOI:** 10.3390/pharmaceutics14101988

**Published:** 2022-09-20

**Authors:** Mifang Li, Yeying Wang, Xinai Han, Yibiao Liu, Mingliang Ma, Lingyan Zhang

**Affiliations:** 1 Department of Medical Imaging, Longgang Central Hospital of Shenzhen, 6082 Longgang Avenue, Longgang District, Shenzhen 518116, China; 2 Medical Frontier Innovation Research Center, The First Hospital of Lanzhou University, Lanzhou 730000, China; 3 Shanghai Engineering Research Center of Molecular Therapeutics and New Drug Development, School of Chemistry and Molecular Engineering, East China Normal University, 3663 N. Zhongshan Road, Shanghai 200062, China; 4 Department of Rheumatology and Immunology, The Third Affiliated Hospital, Southern Medical University, Guangzhou 518048, China

**Keywords:** theranostics, lupus nephritis, multi-mode imaging, collaborative treatment

## Abstract

Lupus nephritis (LN) is a common and refractory inflammation of the kidneys caused by systemic lupus erythematosus. Diagnosis and therapies at this stage are inefficient or have severe side effects. In recent years, nanomedicines show great potential for imaging diagnosis and controlled drug release. Herein, we developed a polydopamine (PDA)-based nanocarrier modified with Fe_3_O_4_ and Pt nanoparticles and loaded with necrostatin-1 (Nec-1) for the bimodal imaging and therapy of LN. Results demonstrate that Nec-1/PDA@Pt-Fe_3_O_4_ nanocarrier exhibits good biocompatibility. Nec-1, as an inhibitor of receptor-interacting protein 1 kinase, can be used to inhibit receptor-interacting protein 1 kinase activity and then reduces inflammation due to LN. Experiments in vitro and in the LN mouse model confirmed that the nanocarrier can reduce neutrophil extracellular traps (NETs) production by RIPK1 and alleviate the progression of inflammation. Previous studies proved that Pt nanoparticles can catalyze H_2_O_2_ to produce oxygen. A blood oxygen graph of mouse photoacoustic tomography confirmed that Nec-1/PDA@Pt-Fe_3_O_4_ can generate oxygen to fight against the hypoxic microenvironment of LN. PDA and Fe_3_O_4_ are used as photographic developers for photoacoustic or magnetic resonance imaging. The preliminary imaging results support Nec-1/PDA@Pt-Fe_3_O_4_ potential for photoacoustic/magnetic resonance dual-mode imaging, which can accurately and non-invasively monitor microscopic changes due to diseases. Nec-1/PDA@Pt-Fe_3_O_4_ combining these advantages exhibited outstanding performance in LN imaging and therapy. This work offers valuable insights into LN diagnosis and therapy.

## 1. Introduction

Lupus nephritis (LN) is a common and refractory inflammation of the kidneys caused by systemic lupus erythematosus (SLE), which occurs within 6–36 months after diagnosis [1]. Diagnostic and therapeutic tools for LN are currently available, but 14–33% of patients fail to respond, and LN remains a refractory disease [2,3,4]. The diagnosis of LN mainly relies on biomarker-based tests in the blood and histological analysis of kidney biopsies [5]. LN is mainly treated through immunosuppressive therapy and treatments with anti-inflammatory drugs [1,6]. Neutrophil extracellular traps (NETs) and the hypoxia microenvironment of the kidney plays a key role in the pathogenic course of LN [7,8,9,10]. The formation of NETs can be induced by receptor-interacting protein kinase 1 (RIPK1), which aggravates inflammation [5,6,7]. NETs are highly expressed in the kidneys of patients with SLE, leading to severe organ damage [11,12]. In addition, sustained inflammation and immune responses are accompanied by an increased oxygen consumption, resulting in hypoxia in local inflammatory tissues, which further drives inflammation and the formation of extracellular traps in LN [8,13]. Immunosuppression therapy is often used to treat LN. However, it does not always provide adequate control of the disease and carries a high risk of treatment intolerance and dose-increasing toxicity, and a high recurrence rate because of adverse pharmacokinetics and biodistribution.

Owing to the enormous potential of imaging diagnosis and controlled drug release, nanomedicines have been applied to biomedicine. The development of diagnostic nanoparticles provides more accurate enhanced images. Therapeutic nanoparticles improve the accumulation and release of drugs at the pathological site, increase the therapeutic efficacy in general, and reduce the incidence and intensity of side effects. The theranostic purpose can be well achieved by integrating the diagnostic and therapeutic drugs into a single NP [14,15,16]. Polydopamine (PDA) has become a subject of great interest in nanomedicine because of its excellent properties, including good biocompatibility, easy preparation, easy modification, and high near-infrared absorption [17,18,19,20]. In addition, PDA can be used as a free radical scavenger because of its rich phenolic groups and eliminates reactive oxygen species (ROS) produced in inflammatory reactions [21,22]. Moreover, PDA is an outstanding photoacoustic imaging contrast agent because of its strong absorption in the near-infrared region [23,24]. Superparamagnetic complexes, such as SPIO, ferric oxide, and Fe_3_O_4_, have been successfully used in MRI to detect LN and other tissue sites of inflammation [25,26]. When injected into the bloodstream, these nanoparticles are absorbed by inflammatory cells and accumulate in inflammatory tissues, reducing signal attenuation on T2-weighted images, which can be used in detecting inflammation [27]. Combinations of PDA and superparamagnetic complexes can be used in visualizing the molecular changes of LN and monitoring microhemodynamic changes in the kidney through PA/MR bimodal imaging. Precious metal platinum (Pt) is used in oxygen production in vivo because it has high catalase catalytic activity, good biocompatibility, and high cell uptake efficiency [28]. In our previous study, we prepared Pt-based nanomaterials that significantly improve the hypoxia microenvironment of tumors by in situ catalytic hydrogen peroxide (H_2_O_2_) [29].

**Figure 1:**
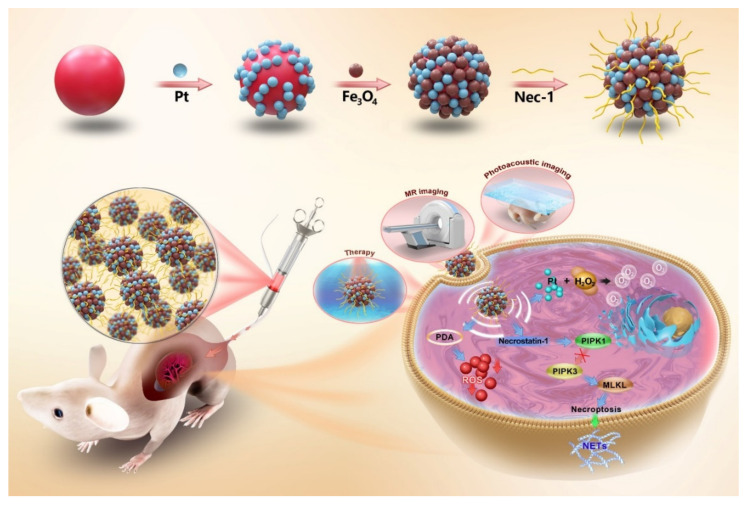
The preparation process of Nec-1/PDA@Pt-Fe_3_O_4_ nanoparticles and schematic illustration of the Nec-1/PDA@Pt-Fe_3_O_4_ nanoparticles for imaging and therapy of LN. Nec-1/PDA@Pt-Fe_3_O_4_ nanoparticles consisted of a polydopamine (PDA)-based nanocarrier modified with Fe_3_O_4_ and platinum (Pt) and loaded with necrostatin-1 (Nec-1) for photoacoustic/magnetic resonance dual-mode imaging and therapy of LN. As a necroptosis inhibitor, Nec-1 selectively targets the kinase activity of receptor-interacting protein kinase 1 (RIPK1), and thus inhibits mixed lineage kinase domain-like (MLKL) phosphorylation. Nec-1 is used to inhibit neutrophil extracellular traps (NETs) release and further reduces inflammation due to LN. PDA can be used as a free radical scavenger and eliminates reactive oxygen species (ROS) produced in inflammatory reactions. Moreover, Pt is used in oxygen production because of its high catalase catalytic activity.

In this work, we prepared PDA-based nanoparticles modified with Fe_3_O_4_ and Pt nanoparticles (PDA@Pt-Fe_3_O_4_) and loaded with necrostatin-1 (Nec-1), an inhibitor of RIPK1, for the bimodal imaging and therapy of LN (Figure 1). Nec-1 is used to inhibit RIPK1 kinase activity and further reduces inflammation due to LN. PDA and Fe_3_O_4_ have been used as photographic developers for photoacoustic/magnetic resonance dual-mode imaging for the accurate and non-invasive monitoring of microscopic changes caused by diseases. Pt nanoparticles are used to alleviate inflammation induced by hypoxia. This work offers valuable insights into LN diagnosis and therapy.

## 2. Experimental Section

### 2.1. Materials

Ammonium hydroxide (NH_3_·H_2_O, 25–28%, *w*/*w*), dopamine, polyvinylpyrrolidone (PVP, K30), hydrogen hexachloroplatinate (H_2_PtCl_6_), ferric chloride hexahydrate (FeCl_3_·6H_2_O), ferrous chloride tetrahydrate (FeCl_2_·4H_2_O) and Nec-1 were purchased from Sigma Aldrich (Shanghai, China). Anhydrous ethanol was obtained from Sinopharm Chemical Reagent Co., Ltd. (Shanghai, China). Phorbol 12-myristate-13-acetate (PMA) was purchased from Beyotime (Wuhan, China). Sytox Green was purchased from Keygen Biotech (Wuhan, China). Phosphate buffered saline (PBS) was obtained from KH_2_PO_4_, Na_2_HPO_4_, hydrochloric acid, sodium hydroxide, and deionized water. All the chemical reagents were of analytical grade and used directly as received. A Milli-Q water purification system (Bedford, MA, USA) was used in obtaining deionized water.

### 2.2. Preparation of Polydopamine (PDA) Nanoparticles

Firstly, 18 mL of ultrapure water and 8 mL ethanol were mixed and stirred gently at 30 °C. Secondly, 0.6 mL of NH_3_·H_2_O was added, and the mixture was stirred for 30 min. Then, 2 mL of dopamine solution (50 mg/mL) and hydrochloride were added [30]. The resulting solution was stirred for 18 h. Finally, the solution was centrifuged at 15,000 rpm for 15 min and washed three times with ultrapure water for the preparation of PDA nanoparticles.

### 2.3. Preparation of PDA@Pt Nanoparticles

PDA@Pt nanoparticles were synthesized by a chemical reduction method [31]. In brief, 10 mg of PDA and 50 mg of PVP were dispersed in 100 mL of ethanol under ultrasonic conditions. Then, 0.8 mL of 120 × 10^−3^ mol/L H_2_PtCl_6_ was added to the above solution, which was then subjected to ultrasonic stirring for 30 min and reflux at 90 °C for 12 h. PDA@Pt nanoparticles were prepared by washing, centrifugation, and dispersion with deionized water.

### 2.4. Preparation of PDA@Pt-Fe_3_O_4_ Nanoparticles

Approximately 15 mg of PDA@Pt nanoparticles were dispersed in 55 mL of deoxidized ultrapure water to form a uniform suspension. Subsequently, 54 mg of FeCl_3_·6H_2_O and 23 mg of FeCl_2_·4H_2_O were added to the suspension. A water bath was heated to 85 °C, and when the temperature was stable, 3.75 mL of NH_3_·H_2_O was added, and stirring was continued for 1 h [32]. The PDA@Pt-Fe_3_O_4_ nanoparticles were prepared by washing several times with ethanol and dispersing them in water.

### 2.5. General Characterization

The morphology of nanoparticles was recorded with high-resolution transmission electron microscopy at an accelerating voltage of 200 kV (JEM-2100, JEOL, Tokyo, Japan). The size and zeta potential of the nanoparticles were determined through dynamic light scattering (DLS) analysis using a Zetasizer Nano ZS equipment (Malvern Instruments Nano ZSE, UK). Each sample was measured five times. Fourier transform infrared spectroscopy (FTIR) spectra were obtained using a TENSOR II spectrometer (Bruker, DER). Crystallinity was measured by powder X-ray diffraction on a D/Max-2550 PC X-ray diffractometer (Rigaku, Japan) with Cu-Ka radiation. UV-Vis-NIR absorption spectra were measured on a Cary 5000 UV-visible spectrophotometer (Agilent, CA, USA). Pathological images were obtained with an upright metallurgical microscope (Carl Zeiss, Axio Imager A2, Jena, Germany). The concentrations of Fe and Pt solutions were measured by inductively coupled plasma atomic emission spectrometry (710-ES, Varian, Palo Alto, CA, USA). Pathological images were obtained with an upright metallurgical microscope (Carl Zeiss, Axio Imager A2, Jena, Germany).

### 2.6. Nec-1 Loading and Release

Nec-1 was loaded on the PDA@Pt-Fe_3_O_4_ nanoparticles by mixing 2 mL of freshly prepared Nec-1 solution (0.5 mg/mL) with 5 mg of PDA@Pt-Fe_3_O_4_ nanoparticles. The mixed solution was stirred in the dark for 48 h at room temperature to reach an equilibrium state. Then, the remaining unbound Nec-1 was wiped off through centrifugation. The loading capacity of Nec-1 was calculated by the following equation:

(1) $$\mathrm{Loading}\;\mathrm{capacity}=\left(\frac{\mathrm{weight}\;\mathrm{of}\;\mathrm{loaded}\;\text{Nec-1}}{\mathrm{weight}\;\mathrm{of}\;\mathrm{nanoparticles}\;\mathrm{and}\;\mathrm{loaded}\;\text{Nec-1}}\right)\times 100\%$$

Nec-1 released from PDA@Pt-Fe_3_O_4_ nanoparticles was tested as follows. Nec-1/PDA@Pt-Fe_3_O_4_ nanoparticles (2.5 mg) were dispersed in 5.0 mL of PBS solution at pH 6.5 and pH 7.4 successively. The solutions were stirred in a shaker (Boxun Instrument Co., Ltd., Changsha, China) at 37 °C and subjected to centrifugation at certain time points. The supernatants were collected, and the Nec-1/PDA@Pt-Fe_3_O_4_ nanoparticles at the bottom were re-dispersed in equal volumes of fresh PBS solutions under ultrasonication. The amounts of released Nec-1 in corresponding PBS supernatants (pH 6.5 and 7.4) at various times were obtained via the characteristic absorption of Nec-1.

### 2.7. Evaluating the Formation of NETs

The PMA-induced formation of NETs in HL-60 cells was investigated, and the amount of the NETs was determined in vitro. The cells were induced to differentiate into neutrophil-like granulocytes by DMSO. Then, neutrophil-like granulocytes (1.5 × 10^6^ cells/well in 200 μL of medium) were seeded into black, flatbottomed, and 96-well plates and incubated for 4 h in a humidified incubator at 37 °C in an atmosphere containing 5% CO_2_. The medium was supplemented with PBS, 50 μg/mL Nec-1/mPDA@Pt-Fe_3_O_4_, and 25 nM PMA. Another medium without 25 nM PMA was used. Extracellular DNA was stained with the membrane-impermeable DNA-binding dye Sytox Green. The plates were analyzed using a Spectra Max M3 fluorescent plate reader (Molecular Devices, San Jose, CA, USA) with excitation at 485 nm and emission at 520 nm [12].

### 2.8. Animal Model Establishment

Female C57BL/6 mice (15 weeks old) were purchased from Guangdong Medical Laboratory Animal Center (Foshan, China). Animal work was performed using the protocols approved by the Institutional Animal Care and Use Committee. Female C57BL/6 mice aged 5–6 weeks were intraperitoneally injected with 0.5 mL of pristane. The urinary protein content of the mice was detected by Albustix dipsticks every week, and the symptoms and signs in the mice were observed. When there were 3 + urine proteins on the paper, and the mice’s paws were pale and cold with ascites, the model was considered successfully established [33].

### 2.9. MR Imaging and Photoacoustic Imaging of the Nec-1/PDA@Pt-Fe_3_O_4_

Nec-1/PDA@Pt-Fe_3_O_4_ at different concentrations (0.8, 0.4, 0.2, 0.1 and 0 mM) was dispersed in 3% agar mixture. All samples were placed in test tubes and scanned with 3.0T MRI with a small animal coil (Philips Healthcare, Best, the Netherlands). The imaging parameters for axial T2-weighted imaging (T2WI) sequence were as follows: relaxation time (TR) = 2408 ms, echo time (TE) = 100 ms, slice thickness = 3.5 mm, slice spacing = 0.35 mm, field of view = 180 mm × 180 mm, matrix = 328 × 251, and scanning time = 82 s.

Nanoprobes with different concentrations (0.1, 0.2, 0.4, and 0.8 mg/mL) were poured into plastic tubes, which were placed in a photoacoustic microscope imager (developed by Guangdong Provincial People’s Hospital) for scanning. The photoacoustic imager had a laser wavelength of 532 nm.

Photoacoustic imaging and MR imaging in vivo were performed on the mice in the model group. After anesthesia administration, lupus mice were placed in a 3.0T MR imager (Philips Healthcare, Best, the Netherlands) and scanned with a 3.0T MRI with a small animal coil (Philips Healthcare, Best, the Netherlands). Nec-1/PDA@Pt-Fe_3_O_4_ nanoprobe (100 μL) was injected into the tail vein of each mouse at a concentration of 1 mg/mL. T2WI was performed at baseline (prior to injection) and 5 min after Nec-1/PDA@Pt-Fe_3_O_4_ injection. The imaging parameters for coronal T2WI sequence were as follows: TR = 2500 ms, TE = 100 ms, slice thickness = 3 mm, slice spacing = 0.3 mm, field of view = 80 mm × 80 mm, matrix = 160 × 119, and scanning time = 65 s.

Lupus mice were scanned by photoacoustic tomography (VisualSonics, Vevo LAZR, Toronto, Canada). The mice were fixed in photoacoustic tomography, and the renal photoacoustic images were collected. A 100 μL Nec-1/PDA@Pt-Fe_3_O_4_ nanoprobe was injected into the tail vein of mice at a concentration of 1 mg/mL. After 5 min, photoacoustic imaging was performed on the kidney again, and the intensities of photoacoustic signals in the kidney region before and after the injection of the nanoprobe were compared.

### 2.10. Therapeutic Effect of Nec-1/mPDA@Pt-Fe_3_O_4_ on LN

LN mice (16 weeks) were randomly divided into three groups, including control, model, and Nec-1/mPDA@Pt-Fe_3_O_4_ groups. Intraperitoneally, Nec-1 nanoprobes were injected at a concentration of 1 mg/mL and a dosage of 6 mg/kg/day. All mice were treated for 3 weeks, their body weights were measured every 3 days and urinary protein excretion was measured every week. They were killed at the end of the experiment. Mouse kidneys were collected and stained with hematoxylin and eosin (H&E), and structural changes in the kidney tissues were observed. Then, myeloperoxidase (MPO; antibodies to neutrophil markers) was used to stain the kidney tissues. Finally, an upright metallographic microscope was used for image acquisition. Differences in treatment outcomes among groups were analyzed by *t*-test.

The renal areas of different groups were scanned with a 3.0T MRI (Philips Healthcare, Best, the Netherlands). The imaging parameters for coronal intravoxel incoherent motion imaging (IVIM-DWI) sequence were as follows: TR = 3000 ms, TE = 61 ms, scanning layer thickness = 2.4 mm, layer spacing = 0.24 mm, field of vision = 70 mm × 66 mm, matrix = 64 × 72, and scanning time = 189 s. IVIM-DWI image post-processing: Philips post-processing workstation was used to analyze the image, delineate the renal parenchyma area of interest, and calculate the perfusion score F value.

Lupus mice were scanned by photoacoustic tomography (VisualSonics, Vevo LAZR, Toronto, Canada). After anesthesia, the mice were fixed in photoacoustic tomography, the renal blood oxygen images of each group were collected, and the renal blood oxygen status of each group was compared with that of another.

### 2.11. Biosafety Assessment of Nec-1/mPDA@Pt-Fe_3_O_4_

Mice were randomly divided into the experimental group and the control group, and the experimental group was injected with Nec-1/mPDA@Pt-Fe_3_O_4_ (1mg/mL, 6mg/kg/d Necrostatin-1 equiv.) via the intraperitoneal injection, and the control group was injected with saline (100 mL). The hearts, livers, spleens, lungs, and kidneys of the mice were collected for histological analysis.

### 2.12. Statistical Analysis

Experimental results were shown as the mean ± standard deviation. All the statistical analyses were performed by Statistical Product and Service Solutions for Windows (SPSS, version 22.0, IBM Co., Ltd., Armonk, NY, USA). The difference between the iVIM-DWI sequence renal perfusion scores and percentage content of NETs released by neutrophils of different groups was analyzed by one-way ANOVA. *p* < 0.05 indicated statistical difference.

## 3. Results and Discussion

### 3.1. Preparation and Characterization of Nec-1/PDA@Pt-Fe_3_O_4_

**Figure 2:**
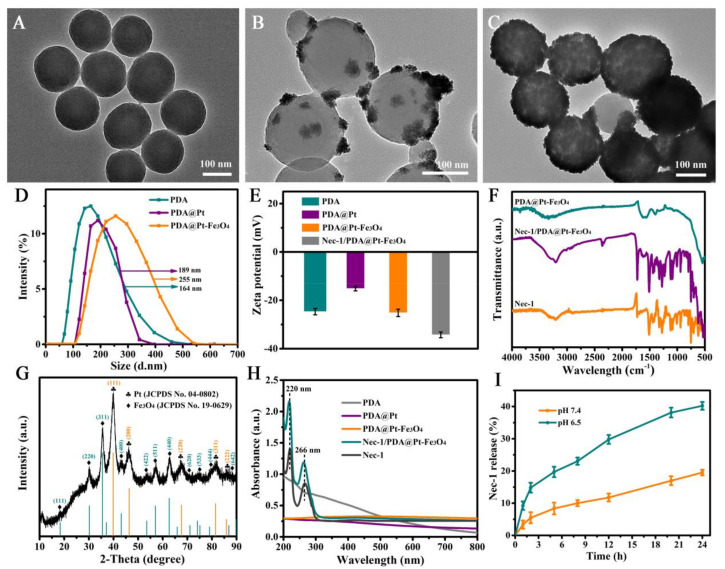
The TEM images of (**A**) PDA nanoparticles, (**B**) PDA@Pt nanoparticles, (**C**) PDA@Pt-Fe_3_O_4_ nanoparticles. (**D**) The dynamic light scattering of PDA, PDA@Pt, and PDA@Pt-Fe_3_O_4_ nanoparticles, respectively. (**E**) The zeta potential of PDA, PDA@Pt, PDA@Pt-Fe_3_O_4,_ and Nec-1/PDA@Pt-Fe_3_O_4_ nanoparticles, respectively. (**F**) FTIR spectra of the PDA@Pt-Fe_3_O_4_, Nec-1/PDA@Pt-Fe_3_O_4_, and free Nec-1. (**G**) XRD pattern of the PDA@Pt-Fe_3_O_4_. (**H**) UV-Vis-NIR spectra of the aqueous solutions of PDA, PDA@Pt, PDA@Pt-Fe_3_O_4_, Nec-1/PDA@Pt-Fe_3_O_4,_ and free Nec-1. (**I**) Cumulative Nec-1 release of the Nec-1/PDA@Pt-Fe_3_O_4_ nanoparticles in PBS at pH 6.5 and 7.4.

The Nec-1/PDA@Pt-Fe_3_O_4_ nanoparticles were designed according to Figure 1. PDA nanoparticles were prepared by an oxidation self-polymerization method. Pt was then coated on the surface of PDA by a chemical reduction method for the preparation of PDA@Pt nanoparticles. Fe^3+^/Fe^2+^ were converted into Fe_3_O_4_ nanoparticles on the surface of PDA@Pt nanoparticles in the presence of ammonium hydroxide. As demonstrated in Figure 2A, the prepared PDA nanoparticles were monodispersed with well-defined spherical shapes and had a homogeneous diameter of ~142 nm. Figure 2B shows that the PDA@Pt nanoparticles were constructed successfully. After Fe_3_O_4_ coating, PDA@Pt-Fe_3_O_4_ formed (Figure 2C). The results provided indirect proof of the success of each step. We measured the hydrated particle sizes of PDA, PDA@Pt, and PDA@Pt-Fe_3_O_4_ by DLS (Figure 2D,E), and the average diameters were ~164, ~189, and ~255 nm, which were slightly larger than the corresponding TEM results because DLS showed an average hydrodynamic particle size. Subsequently, the zeta potentials of these nanoparticles were investigated (Figure 2E). The zeta potentials of PDA were negative on account of the existence of phenolic hydroxyl groups on the surface. The zeta potential of PDA@Pt increased to accommodate the negative hydroxyl in PDA. The zeta potential value of PDA@Pt-Fe_3_O_4_ decreased from −15.2 mV to −25.3 mV, which may be attributed to the successful Fe_3_O_4_ coating. The zeta potential of Nec-1/PDA@Pt-Fe_3_O_4_ decreased to −34.6 mV, which may be due to the ionization of Nec-1. These results showed that Nec-1/PDA@Pt-Fe_3_O_4_ had been successfully prepared.

To verify the encapsulation of Nec-1, we compared the FTIR spectra of PDA@Pt-Fe_3_O_4_, Nec-1/PDA@Pt-Fe_3_O_4_, and free Nec-1. As shown in Figure 2F, the characteristic peak at 512 cm^−1^ in the spectra of PDA@Pt-Fe_3_O_4_ should be assigned to the Fe–O bond vibration of Fe_3_O_4_ nanoparticles. Compared with the characteristic peaks of Nec-1, Nec-1/PDA@Pt-Fe_3_O_4_ was successfully prepared. Subsequently, the X-ray diffraction pattern of PDA@Pt-Fe_3_O_4_ is presented in Figure 2G. The characteristic peaks of Pt nanocrystals (JCPDS No. 04-0802) and Fe_3_O_4_ nanocrystals (JCPDS No. 19-0629) demonstrated that Pt nanocrystals and Fe_3_O_4_ were successfully assembled in the final nanoparticles.

Figure 2H shows the UV-Vis-NIR absorption spectra of PDA, PDA@Pt, PDA@Pt-Fe_3_O_4_, Nec-1/PDA@Pt-Fe_3_O_4_, and free Nec-1. The aqueous solution of PDA, PDA@Pt, and PDA@Pt-Fe_3_O_4_ revealed no obvious absorption peaks. The distinct characteristic absorption peak at 220 and 266 nm of Nec-1 appeared in the spectra of Nec-1/PDA@Pt-Fe_3_O_4_, indicating that Nec-1 was successfully loaded in the Nec-1/PDA@Pt-Fe_3_O_4_ nanoparticles. In addition, we calculated the loading capacity of Nec-1. As indicated by the UV-Vis-NIR spectra of PDA@Pt-Fe_3_O_4_ and Nec-1, the Nec-1 loading capacity in Nec-1/PDA@Pt-Fe_3_O_4_ nanoparticles was 17% (by weight). These results showed that we successfully prepared Nec-1/PDA@Pt-Fe_3_O_4_ nanoparticles.

Further, the Nec-1 release properties of Nec-1/PDA@Pt-Fe_3_O_4_ nanoparticles were studied in PBS with pH 6.5 (simulated LN lesion environment) and pH 7.4 (simulated physiological condition), respectively. The results (Figure 2I) showed that 40.2% of Nec-1 was released under pH 6.5 within 24 h, much higher than that at pH 7.4, which is due to the fact that the -HC=N- bonds on the surface of nanoparticles can be disrupted in an acidic lupus nephritis environment, resulting in the specific release of Nec-1 at the lesion site [34]. The results demonstrated that the Nec-1 release from Nec-1/PDA@Pt-Fe_3_O_4_ nanoparticles can be controlled by pH. The high release is beneficial for the treatment of lupus nephritis as it rapidly produces effective local Nec-1 concentrations.

### 3.2. PA and MR Dual-Mode Image Tracking

**Figure 3:**
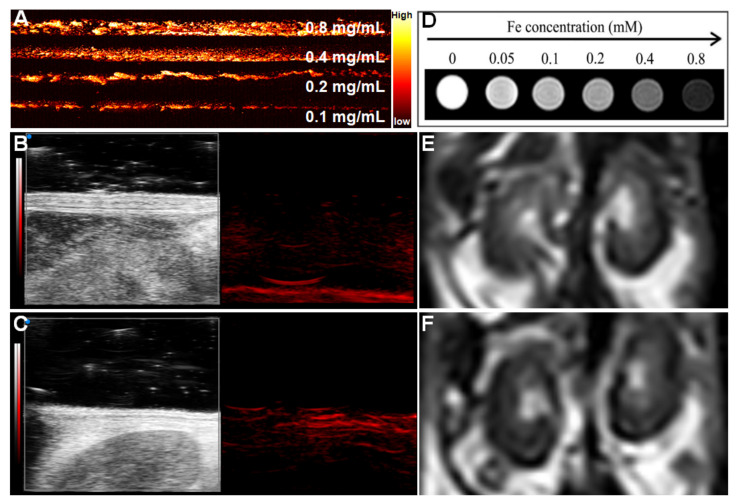
Photoacoustic/magnetic resonance dual-mode imaging. (**A**) Photoacoustic imaging at different concentrations of Nec-1/PDA@Pt-Fe_3_O_4_ in vitro, Photoacoustic imaging of mice kidney before (**B**) and after (**C**) adding 1mg/mL Nec-1/PDA@Pt-Fe_3_O_4_ in vivo. (**D**) T_2_-weighted magnetic resonance imaging in vitro at different concentrations of Nec-1/PDA@Pt-Fe_3_O_4_. Magnetic resonance imaging of mice kidneys before (**E**) and after (**F**) adding Nec-1/PDA@Pt-Fe_3_O_4_ in vivo.

To explore the PA performance of Nec-1/PDA@Pt-Fe_3_O_4_, we used a variable wavelength pulsed laser (680–970 nm) and an input laser with a frequency of 30 MHz. The results showed that PDA@Pt-Fe_3_O_4_ exhibited extremely strong PA signal intensity, and the intensity of the photoacoustic signal increased with PDA@Pt-Fe_3_O_4_ concentration (Figure 3A) possibly because of the PA-enhancing effect of PDA [23]. In addition, the photoacoustic image of mouse kidneys before and after injection of 100 μL Nec-1/PDA@Pt-Fe_3_O_4_ at a concentration of 1 mg/mL was investigated. The results are shown in Figure 3B,C. After the injection of nanoprobes, the photoacoustic signal of blood vessels in the renal region was significantly enhanced, and the renal contour became clear, indicating that Nec-1/PDA@Pt-Fe_3_O_4_ had an excellent performance in PA images.

The MRI performance of Nec-1/PDA@Pt-Fe_3_O_4_ was investigated with 3.0T MRI. The detailed parameters are shown in the Methods section. As shown in Figure 3D, the T2-weighted signal intensity decreased gradually with increasing concentration. We also investigated MRI performance in vivo. As shown in Figure 3E,F, after Nec-1/PDA@Pt-Fe_3_O_4_ was injected into the mice in vivo, the T2-weighted signal was negatively enhanced.

The above results showed that Nec-1/PDA@Pt-Fe_3_O_4_ is an outstanding PA/MR dual-mode imaging agent.

### 3.3. Therapeutic Effect of Nec-1/PDA@Pt-Fe_3_O_4_ on LN

**Figure 4:**
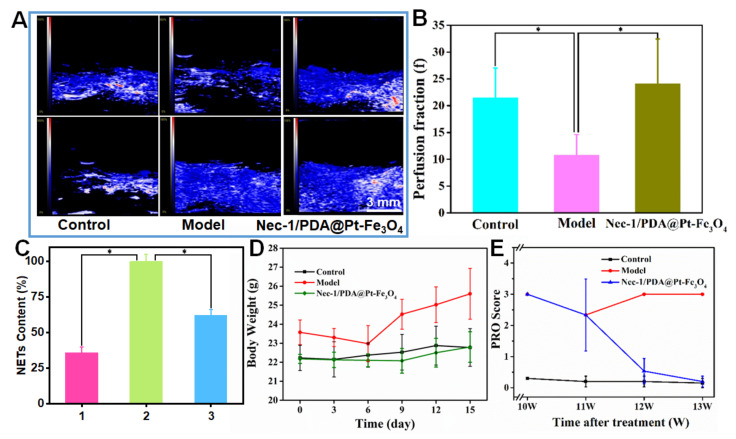
(**A**) Blood oxygen graph of mouse photoacoustic tomography. (**B**) Comparison of iVIM-DWI sequence renal perfusion scores in different groups of mice. Data are presented as the mean ± SD. * *p* < 0.05 vs. the model group. (**C**) The percentage content of NETs released by a neutrophil. 1 represents neutrophil control group; 2 represents neutrophil + PMA; 3 represents neutrophil + PMA + Nec-1/PDA@Pt-Fe_3_O_4_. Data are presented as the mean ± SD. * *p* < 0.05 vs. the neutrophil + PMA group. (**D**) Body weight and (**E**) PRO content change before and after Nec-1/PDA@Pt-Fe_3_O_4_ treatment.

Nec-1/PDA@Pt-Fe_3_O_4_ showed considerable therapeutic quality apart from acting as a dual-mode imaging contrast agent. PDA, as the main material of this nanocomposite, is a good free radical scavenger because of its rich phenolic groups, which eliminates the ROS produced in inflammatory reactions [21]. Pt has a high catalase catalytic activity and can thus catalyze hydrogen peroxide and produce oxygen gas, which significantly improves the hypoxia microenvironment of LN and alleviate LN to a certain extent [35]. To intuitively confirm the change in oxygen level in the kidney area, we carried out a blood oxygen graph of mouse photoacoustic tomography. As shown in Figure 4A, the closer the color level is to 100%, the higher the blood oxygen saturation (SaO_2_) is. The results demonstrated that the SaO_2_ level of the model group greatly decreased relative to that in the control group, indicating that inflammation reduced the oxygen content. After the addition of Nec-1/PDA@Pt-Fe_3_O_4_, the SaO_2_ level in the kidney considerably increased relative to that in the model group and was equal to the SaO_2_ level of the control. These results showed that improving the anoxic microenvironment has a certain therapeutic effect on LN. Moreover, iVIM-DWI sequence renal perfusion scores among the control, model, and drug group were compared (Figure 4B). The results demonstrated that the renal perfusion score of the mice in the model group was lower than that in the control group, indicating that the renal capillaries in the model group mice were damaged. After the injection of Nec-1/PDA@Pt-Fe_3_O_4_, the renal perfusion score of mice significantly increased, and the renal capillaries damage was reduced. This result demonstrated that Nec-1/PDA@Pt-Fe_3_O_4_ decreased inflammation in the kidney and alleviated LN.

Nec-1 is an inhibitor of receptor-interacting protein 1 kinase, which inhibits RIPK1 kinase activity and reduces LN inflammation [36,37]. As shown in Figure 4C, PMA can promote NET production. After the addition of Nec-1/PDA@Pt-Fe_3_O_4_, the NET content decreased to 50%, which demonstrated that Nec-1/PDA@Pt-Fe_3_O_4_ loading Nec-1 had excellent performance in inhibiting RIPK1 kinase activity and reduced NET production by RIPK1. Body weight change in the mice was recorded in the entire experiment. The result showed that the body weight of LN mice (model group) exhibited decreased and then increased. The possible principal reason for this trend was the production of ascites when LN was aggravated. In the control and drug groups, the body weights of the mice were basically stable, suggesting that Nec-1/PDA@Pt-Fe_3_O_4_ can treat LN to some extent (Figure 4D). Furthermore, we measured the urinary protein content. As shown in Figure 4E, the urine protein content of the control group mice remained low, and the urine protein content in the model group mice remained high. After treatment with Nec-1/PDA@Pt-Fe_3_O_4_, the content of urinary protein gradually reduced with the extension of treatment time, demonstrating that the Nec-1/PDA@Pt-Fe_3_O_4_ improved renal function.

**Figure 5:**
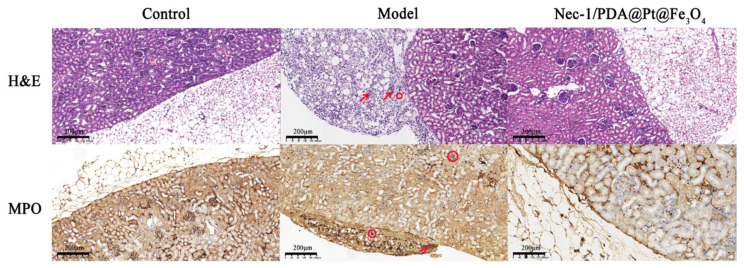
H&E and MPO staining images of kidney sections. The area where the red arrow is pointing represents neutrophils. The red circle area represents lymphocytes.

To further test the therapeutic efficacy of the Nec-1/PDA@Pt-Fe_3_O_4_ for LN, we performed a pathological examination on the kidneys of the mice. The kidneys were sectioned and stained with H&E and MPO. As shown in Figure 5, H&E staining showed numerous neutrophils and lymphocytes that infiltrated the renal cortex, indicating severe kidney inflammation. In the control and drug groups, clear infiltration around the renal cortex without inflammatory cell infiltration was observed, demonstrating that Nec-1/PDA@Pt-Fe_3_O_4_ reduced inflammation and then ameliorated LN. MPO staining showed similar results. Neutrophils and some lymphocytes were observed around the renal cortex in the model group. In the control and drug groups, neutrophils and some lymphocytes around the renal cortex markedly decreased, showing that Nec-1/PDA@Pt-Fe_3_O_4_ can reduce inflammation. In general, these results suggested that Nec-1/PDA@Pt-Fe_3_O_4_ can ameliorate LN.

### 3.4. Biosafety Assessment of Nec-1/PDA@Pt-Fe_3_O_4_

**Figure 6:**
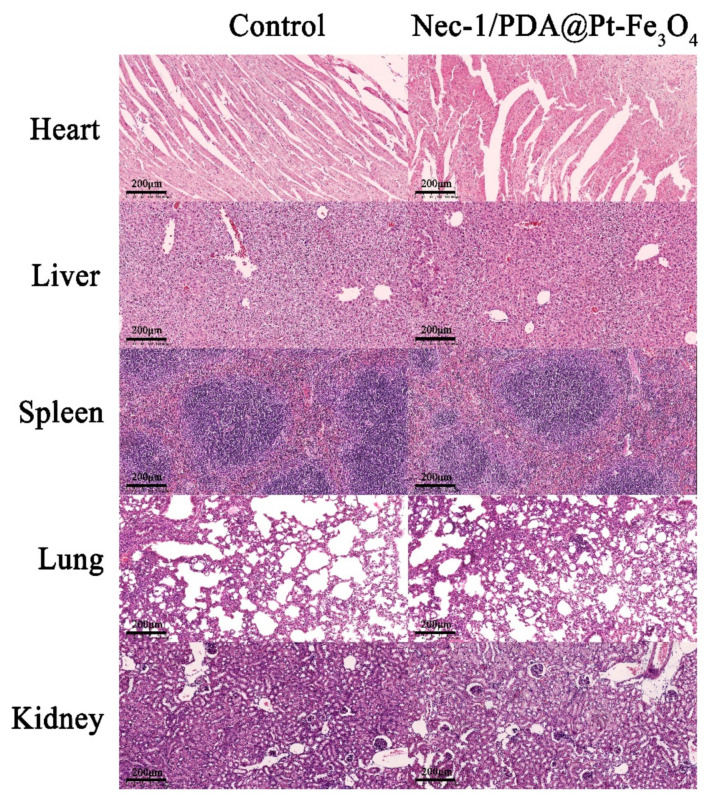
Biosafety and toxicity in vivo. Histological toxicity evaluation of Nec-1/PDA@Pt-Fe_3_O_4_. Organs mainly include the heart, liver, spleen, lungs, and kidney.

To further investigate the potential for clinical applications, we studied the biosafety of Nec-1/PDA@Pt-Fe_3_O_4_ in vivo and performed H&E staining on vital organs (heart, liver, spleen, lungs, and kidney). The results showed no apparent abnormality or damage of mouse vital organs in the histopathological experiment after the mice were treated with Nec-1/PDA@Pt-Fe_3_O_4_ (Figure 6), indicating that Nec-1/PDA@Pt-Fe_3_O_4_ has low toxicity in vivo. Hence, Nec-1/PDA@Pt-Fe_3_O_4_ may be used clinically for LN treatment.

## 4. Conclusions

A multifunctional photoacoustic/magnetic resonance dual-mode-imaging nanocarrier loading Nec-1 was reported for the diagnosis and therapy of LN. This nanocarrier (Nec-1/PDA@Pt-Fe_3_O_4_) consisted of a PDA-based nanocarrier modified with Fe_3_O_4_ and platinum nanoparticles and Nec-1. Nec-1/PDA@Pt-Fe_3_O_4_ are regarded as safe, and we did not observe any toxicities in mice after they received the nanocarrier. The blood oxygen graph of mouse photoacoustic tomography confirmed that Nec-1/PDA@Pt-Fe_3_O_4_ can generate oxygen to fight against the hypoxic microenvironment of LN. Cellular experiments and in vivo tests in the LN mice model confirmed that the nanocarrier can inhibit RIPK1 kinase activity and reduce NET production by RIPK1. After three weeks of systemic administration, the Nec-1/PDA@Pt-Fe_3_O_4_ prominently alleviated the progression of inflammation in the LN mice model. Additionally, the preliminary imaging results confirmed that Nec-1/PDA@Pt-Fe_3_O_4_ can exhibit strong PA signal intensity and a negative enhancement of T2-weighted signal intensity, supporting its potential for photoacoustic/magnetic resonance dual-mode imaging, which can monitor accurately and non-invasively the progression of LN. Nec-1/PDA@Pt-Fe_3_O_4_ combining these benefits exhibited excellent performance in LN diagnosis and therapy. Therefore, Nec-1/PDA@Pt-Fe_3_O_4_ is a promising nanocarrier system for dual-mode imaging and LN therapy. Last, it remains to be explored the full extent of Nec-1/PDA@Pt-Fe_3_O_4_ efficacy in LN mouse models, including, but not limited to, survival analysis, dose-dependent response, the presence of side effects, and the study of the mechanisms involved. We propose that the results from this study establish the translational potential of humans, but further studies should be conducted to prove this hypothesis.

## Data Availability

Not applicable.

## Abbreviations

PDA: polydopamine; Pt: platinum; Fe_3_O_4_: ferroferric oxide; Nec-1: necrostatin-1; LN: lupus nephritis; PMA: Phorbol 12-myristate-13-acetate; RIPK1: receptor interacting protein kinase 1; NETs: neutrophil extracellular traps; ROS: reactive oxygen species; MRI: magnetic resonance imaging; PA: photoacoustic imaging.
